# Cholinergic receptor binding in unimpaired older adults, mild cognitive impairment, and Alzheimer’s disease dementia

**DOI:** 10.1186/s13195-021-00954-w

**Published:** 2022-02-07

**Authors:** David L. Sultzer, Aaron C. Lim, Hailey L. Gordon, Brandon C. Yarns, Rebecca J. Melrose

**Affiliations:** 1 Psychiatry/Mental Health Service, VA Greater Los Angeles Healthcare System, Los Angeles, CA USA; 2 Department of Psychiatry and Human Behavior, School of Medicine, and Institute for Memory Impairments and Neurological Disorders (UCI MIND), University of California, Irvine, Irvine, CA USA; 3 Department of Family Medicine, USC Keck School of Medicine, Alhambra, CA USA; 4 Biomedical Engineering Department, Carnegie Mellon University, Pittsburgh, PA USA; 5 Department of Psychiatry and Biobehavioral Sciences, David Geffen School of Medicine at UCLA, Los Angeles, CA USA

**Keywords:** Cholinergic receptors, Mild cognitive impairment, Alzheimer’s disease, Cognitive aging

## Abstract

**Background:**

Cholinergic neurotransmitter system dysfunction contributes to cognitive impairment in Alzheimer’s disease and other syndromes. However, the specific cholinergic mechanisms and brain structures involved, time course of alterations, and relationships with specific cognitive deficits are not well understood.

**Methods:**

This study included 102 older adults: 42 cognitively unimpaired (CU), 28 with mild cognitive impairment (MCI), and 32 with Alzheimer’s disease (AD) dementia. Each participant underwent a neuropsychological assessment. Regional brain α4β2 nicotinic cholinergic receptor binding (*V*_*T*_/*fp*) was measured using 2-[^18^F]fluoro-3-(2(S)azetidinylmethoxy)pyridine (2FA) and PET imaging. Voxel-wise analyses of group differences were performed. Relationships between receptor binding and cognition, age, and cholinesterase inhibitor medication use were assessed using binding values in six prespecified regions of interest.

**Results:**

SPM analysis showed the group *V*_*T*_/*f*_*p*_ binding differences in the bilateral entorhinal cortex, hippocampus, insula, anterior cingulate, thalamus, and basal ganglia (*p* < .05, FWE-corrected). Pairwise comparisons revealed lower binding in the AD group compared to the CU group in similar regions. Binding in the entorhinal cortex was lower in the MCI group than in the CU group; binding in the hippocampus was lower in the AD group than in the MCI group. AD participants taking cholinesterase inhibitor medication had lower 2FA binding in the bilateral hippocampus and thalamus compared to those not taking medication. In the CU group, age was negatively associated with 2FA binding in each region of interest (*r*_*s*_ = − .33 to − .59, *p* < .05 for each, uncorrected). Attention, immediate recall, and delayed recall scores were inversely associated with 2FA binding in most regions across the full sample. In the combined group of CU and MCI participants, attention was inversely associated with 2FA binding in most regions, beyond the effect of hippocampal volume.

**Conclusions:**

Nicotinic cholinergic receptor binding in specific limbic and subcortical regions is lower in MCI and further reduced in AD dementia, compared to CU older adults, and is related to cognitive deficits. Cognitive decline with age may be a consequence of reduced cholinergic receptor density or binding affinity that may also promote vulnerability to other Alzheimer’s processes. Contemporary modification of the “cholinergic deficit” of aging and AD may reveal opportunities to prevent or improve clinical symptoms.

## Introduction

Acetylcholine neurotransmitter system dysfunction has been observed across the continuum from cognitive aging to mild cognitive impairment (MCI) and Alzheimer’s disease (AD) [1–6]. Beginning in the 1970s, AD has been associated with a loss of cholinergic neurons in the nucleus basalis of Meynert and their broad cortical projections [7, 8], as well as choline acetyltransferase decline in the cortex [9].

More recent studies indicate that atrophy of basal forebrain cholinergic neurons occurs with normal aging and accelerates after age 65 years, with further volume loss in early AD dementia [10]. Importantly, cholinergic receptors in the hippocampus and entorhinal cortex that modulate cellular, synaptic, and network activity in learning and memory processes are particularly susceptible to afferent cholinergic loss [11–13]. Further support for the role of the cholinergic system in cognition comes from observations that cholinergic antagonists worsen attention and memory, whereas pro-cholinergic treatments provide modest symptomatic improvement in patients with AD dementia [6, 14].

Relationships between cholinergic system dysfunction and other AD pathologies are complex and bidirectional. For example, cholinergic receptors may be adversely affected by beta amyloid peptides [15], but cholinergic neurotransmission also influences amyloid processing [16, 17] and may protect neurons from amyloid toxicity [18, 19]. Tau pathology is prominent in the basal forebrain early in the AD process [4] and may initiate distant cortical degeneration [12, 20]. There are additional complex links between cholinergic system dysfunction and tau processing [21–23], neuroinflammation [18, 24], cortical volume loss [25, 26], and apolipoprotein E ε4-mediated neuronal alterations in the medial temporal cortex [27]. Cholinergic receptor activity also modulates other neurotransmitter systems and may support synaptic integrity via the glutamatergic pathway in the hippocampus [28]. However, despite major recent advances in understanding the cellular and molecular events in AD, the sequence of evolving pathophysiologies, relationships with clinical symptoms, and value as therapeutic targets are incompletely understood. Whether cholinergic system dysfunction occurs early, late, or across the continuum from cognitive aging to AD remains unclear [3, 6, 22].

Evaluating cholinergic neurotransmission in vivo in cognitive aging and AD can improve the understanding of AD pathophysiology, reveal the role of cholinergic dysfunction in the cascade, and identify treatment targets or biomarkers for treatment response. Relationships between cholinergic dysfunction and cognitive impairment seen in previous studies suggest that cholinergic interventions may help to improve clinical symptoms as well as ameliorate the toxic neurodegenerative cascade. Neuroimaging with cholinergic receptor ligands has been used to measure receptor binding in AD and links to cognitive abilities [29–34]. Our group used 2-[18F]fluoro-3-(2(S)azetidinylmethoxy)pyridine (2FA) and positron emission tomography [35–37] to measure α4β2 nicotinic cholinergic receptors (nAChR) in vivo in AD [38]. In our study, 2FA binding was lower in the medial temporal cortex, anterior cingulate, insula/opercula, medial thalamus, inferior caudate, and brainstem in patients with mild to moderate AD compared to cognitively unimpaired older adults, although binding was not associated with cognition in the AD participants. However, across the studies to date, sample sizes have been relatively small, results have varied, and few have evaluated cholinergic receptor binding across the spectrum of aging, MCI, and clinical AD.

The current cross-sectional study used 2FA PET imaging to measure nicotinic receptor binding in cognitively unimpaired older adults and individuals with MCI and AD dementia. The goal of the study was to compare the regional binding profiles across this continuum, with particular attention to binding in the medial temporal cortex where alterations may have prominent cognitive consequences. A second goal was to assess the relationships between regional nAChR binding and attention, concentration, and memory skills.

## Materials and methods

### Participants

Study participants (*n* = 102) included cognitively unimpaired older adults (CU; *n* = 42), individuals with MCI (*n* = 28), and those with AD dementia (*n* = 32). A subset of participants (CU: *n* = 22; AD: *n* = 24) was included in a prior study by our group [38]. Participants were recruited from VA Greater Los Angeles Healthcare System (VAGLAHS) specialty clinics that assess or treat memory disorders, as well as primary care sites. Additional participants were recruited from community programs. All participants were age 60 years or older. The exclusion criteria included the following: history of tobacco use in the past 10 years, history of head trauma with loss of consciousness for more than 30 min, a history of neurological disorder such as seizure disorder, movement disorder, or stroke, and a history of primary psychotic disorder or bipolar disorder. Individuals currently taking antidepressants (*n* = 27), cholinesterase inhibitor medication (AChEI; *n* = 32), or memantine (*n* = 20) at a stable dosage for at least 3 months were eligible for the study. Those taking other psychotropic medications were excluded.

### Study procedures

All participants underwent a research clinical assessment that included medical history interview, review of cognitive symptoms and functional abilities, review of prior clinical neuroimaging studies, and neuropsychological assessment. The results were reviewed by two neuropsychologists to confirm study eligibility and to assign a diagnostic group (CU, MCI, or AD). Neuropsychological test scores (see below) were converted to *z*-scores based on published norms. Performance within each cognitive domain was assessed. If a participant scored at least 1.5 standard deviations below the adjusted norm on two or more tests, the domain was rated as impaired. Estimates of premorbid functioning were considered when making these determinations. The ability to perform instrumental activities of daily living (IADLs) was evaluated using the Lawton and Brody IADL assessment [39] and medical records. Individuals were rated as CU if all cognitive domains showed no impairment and IADLs were performed independently. MCI included those with cognitive impairment in the memory domain and intact IADLs. Participants were diagnosed with probable AD according to the NIA/AA criteria [40] if there was an impairment in at least two cognitive domains (one of which had to be memory) detected through a combination of patient/informant report and objective assessment, insidious symptom onset, and IADL dependence. Amyloid status was not assessed, and the A/T/N classification system was not applied [41]. Following the research assessment, additional exclusion criteria applied to all participants included the following: history or exam suggestive of an alternative dementia diagnosis, evidence of stroke or moderate/severe cerebrovascular disease on structural imaging, or systemic illness, neurologic illness, or medication that could explain the cognitive decline.

### Neuropsychological assessments

The Mini-Mental State Exam (MMSE) and Mattis Dementia Rating Scale were used to assess global cognition. Simple attention was evaluated with the Trails A test and Digit Span. Verbal memory was assessed using the Logical Memory (LM) subtest of the Weschler Memory Scale-Revised and the immediate and delayed free recall tasks of the Consortium to Establish a Registry for Alzheimer’s Disease (CERAD) word-list memory test. Visuospatial memory was assessed using the Brief Visuospatial Memory Test-Revised (BVMT-R) and the recall condition of the Rey-Osterrieth (Rey O) Complex Figure task.

Three average scores were generated: (1) an average attention score (ATT) was generated by averaging *z*-scores from the Stroop Word Reading, Stroop Color Naming, and Trails A tasks; (2) an average immediate recall (IR) score was generated by averaging across the three learning-trials *z*-scores in the CERAD, BVMT-R, and Logical Memory I tests; and (3) an average delayed recall (DR) score was established by averaging across *z*-scores from the CERAD delayed memory, BVMT-R delayed recall, Rey-O delayed recall, and Logical Memory II tests.

### Magnetic resonance imaging (MRI)

We obtained structural brain MR images using a fast 3D-MPRAGE sequence (T1; TR, 2000; TE, 2.59; TI, 900; slice thickness 1.0mm) on a 1.5-T Magnetom Symphony System scanner (Siemens, Washington, DC). The images were used to help define anatomic regions and to generate gray matter maps for use in the 2FA PET imaging analyses. Hippocampal volume, proportional to intracranial cavity volume, was calculated for each participant using FreeSurfer (http://surfer.nmr.mgh.harvard.edu/) [42].

### 2FA PET imaging

The 2FA radiotracer utilized in this study was prepared at the VAGLAHS Cyclotron Facility [43]. Procedures for the radiotracer synthesis and infusion, image acquisition, and plasma analyses have been described previously [35, 44]. Briefly, study participants received an initial intravenous bolus infusion of 143.6 (SD = 12.6) MBq 2FA in 5 mL saline, followed by a continuous slow infusion of 145.8 (SD = 8.0) MBq 2FA over the following 3-h tracer uptake and 1-h PET imaging periods. This procedure maintains a stable 2FA level in the brain tissues during PET image acquisition [35, 45, 46].

PET images were acquired using the Gemini TF PET-CT scanner (Philips, Amsterdam, The Netherlands) in three-dimensional mode. Total scan time was 60 min, and images were reconstructed in 1-min frames using a three-dimensional reconstruction algorithm and Gaussian filtered back projection with a kernel of 8 mm full width at half maximum (FWHM).

### Venous plasma assay

Blood samples were collected at the start of PET image acquisition and at 15-min intervals during the 60-min PET acquisition period. Parent (non-metabolized) 2FA in the blood plasma samples was measured using solid-phase extraction [47], and the proportion of unbound 2FA in the plasma was measured after ultrafiltration [48]. 2FA binding volume of distribution (*V*_*T*_/*f*_*p*_) on PET images was calculated using the values of parent and unbound 2FA activity in the plasma. This method accounts for inter-participant variability in radiotracer metabolism and protein binding. *V*_*T*_/*f*_*p*_ approximates nicotinic receptor binding potential and is proportional to *B*_max_/*K*_*d*_ [49].

### PET image processing

2FA PET images were processed and analyzed using SPM12. The 60 1-min frames were motion-corrected and then averaged to create one mean subject-level image. Counts within the image voxels were converted to *V*_*T*_/*f*_*p*_ values by dividing the voxel activity by the activity of parent and unbound 2FA activity in plasma.

### PET image processing and partial volume adjustment

Due to the age of participants and potential cortical atrophy, partial volume correction was applied to the PET images using PETPVE from the SPM Toolbox to normalize 2FA grey matter activity [50]. PETPVE outputs two types of data available for analysis: voxel-wise images (PVE-c) [51] and region-of-interest-based data using a geometric transfer matrix method (PVEc-GTM) [52]. First, MRI and 2FA PET images were co-registered, and MRI images were segmented into white matter (WM), gray matter (GM), and cerebrospinal fluid (CSF) components using the VBM8 toolbox [53]. GM voxels were then spatially weighted as the true value of radioactive isotope uptake. These weighted values were then compared to WM and CSF spatial values, based on the point spread function of the PET tomograph. The three weighted values were used to correct for spill-over of activity in the GM and surrounding tissue [50]. PET images were normalized to MNI space, yielding a 2FA PET image in MNI space that had been corrected for partial volume effects. These images were subsequently smoothed using an 8-mm FWHM kernel and used in subsequent voxel-based analyses.

In the second arm of PETPVE, PVEc-GTM, an MNI space region-of-interest (ROI) atlas (Desikan-Killiany [54]) was manipulated into the participant’s brain space. The atlas was then limited to GM-corrected segmented tissue. The mean voxel 2FA activity was then extracted from six bilateral anatomical ROIs that had shown CU-AD group differences in a prior study [38]: entorhinal cortex, hippocampus, insula, anterior cingulate, thalamus, and caudate (core ROIs). The mean 2FA *V*_*T*_/*f*_*p*_ in the six core ROIs were used in subsequent ROI analyses.

### Statistical analyses

2FA binding differences in the CU, MCI, and AD groups were first compared using a one-way ANOVA in SPM12. The groups were modeled assuming independence and unequal variance, thresholding was used to remove spurious 2FA signal, and implicit masking was applied. The main effects were assessed using a voxel-level significance of *p* < .05 corrected using the familywise error procedure (FWE). Planned post hoc tests compared 2FA binding pairwise between the groups at *p* < .05, FWE-corrected. In addition, we used small volume correction to assess the differences in the medial temporal lobe structures, specifically the bilateral entorhinal cortex and hippocampus. A mask of the bilateral entorhinal and hippocampal regions was created using the neuromorphetrics atlas in SPM (neuromorphometrics.com). Findings within the mask were assessed at the voxel level, *p* <.05, FWE-corrected.

Region-of-interest (ROI) analyses were also conducted to assess the 2FA binding differences among the diagnostic groups in specific anatomical regions. Atrophy-corrected *V*_*T*_/*f*_*p*_ values were extracted from the six bilateral core ROIs. ROI group analyses were conducted using MANOVAs in SPSS v25, with 2FA binding in each region as the dependent variables and group as the independent variable. Bonferroni-corrected post hoc tests explored the pairwise group differences in those regions with a significant overall group effect.

Multiple tests were conducted to ensure that assumptions for MANOVA analyses were met. Mahalanobis distance was calculated to detect multivariate outliers, and one case was removed from further analysis. The Shapiro-Wilks tests were used to examine the normality of each dependent variable for each diagnostic group (*p* = .09–.98). Box’s *M* test was calculated to assess the equality of covariance (Box’s *M* = 224.50, *p* = .07). A priori power calculation for MANOVA indicated a required total sample of 81 to detect an effect size (f2V) of .16 with 80% power, with alpha set at .05, 3 total groups, and 12 response variables. A priori power calculation for correlational analyses indicated a required total sample of 82 to detect an effect size of 0.3 with 80% power, with alpha set at .05 using a two-tailed test. Power calculations were carried out using GPower 3.1.7 [55].

## Results

Sample characteristics and clinical assessment scores for participants in the CU, MCI, and AD diagnostic groups are shown in Table 1. The mean MMSE scores were 27.3 and 19.8 in the MCI and AD groups, respectively. The groups were not significantly different in the distribution of race or years of education. There was a significant difference in age and sex, such that those in the AD group were older and more likely to be male than those in the MCI and CU groups (*p*s < .02). Overall, there was a high proportion of male participants in the sample, as the study primarily recruited veterans. AD participants had received this diagnosis an average of 4.8 years (SD 3.44; range < 1 to 14 years) prior to the time of the 2FA scan.

**Table 1:** Participant characteristics

	Cognitively unimpaired (***n*** = 42)	Mild cognitive impairment (***n*** = 28)	Alzheimer’s disease (***n*** = 32)	Statistics
Male/female	28/14	24/4	32/0	Fisher’s exact test, *p* < .001
Age, years	72.1 (7.5)	74.6 (8.5)	79.4 (7.6)	*F*(2,99) = 7.97, *p* = .001
Education, years	16.1 (2.7)	15.4 (2.5)	14.8 (3.5)	*F*(2,98) = 1.64, *p* = .20
Race/ethnicity, *n*				Fisher’s exact test, *p* = .75
African-American	9	8	9	
White, non-Hispanic	30	16	21	
Hispanic	2	2	2	
Asian American/Pacific Islander	1	2	0	
Duration of dementia, years	–	–	4.8 (3.4)	
MMSE	29.3 (1.1)	27.3 (1.9)	19.8 (5.0)	*F*(2,99) = 93.06, *p* < .001
Dementia Rating Scale total	140.0 (3.65)	133.1 (7.6)	104.5 (24.0)	*F*(2,97) = 60.30, *p* < .001
Current medications, *n* (%)				
Cholinesterase inhibitor	1 (2%)	8 (29%)	23 (72%)	Fisher’s exact test, *p* < .001
Memantine	0 (0%)	3 (11%)	17 (53%)	Fisher’s exact test, *p* < .001
Antidepressant	6 (14%)	9 (32%)	12 (38%)	Fisher’s exact test, *p* < .001
Trails A, s	39.5 (12.2)	48.3 (16.7)	102.6 (62.0)	*F*(2,92) = 28.62, *p* < .001
Attention average *Z*-score	− .80 (.61)	− 1.36 (.61)	− 2.37 (1.19)	*F*(2,92) = 29.25, *p* < .001
Immediate Memory average *Z*-score	− .16 (.62)	− 1.30 (.75)	− 2.30 (.52)	*F*(2,92) = 40.99, *p* < .001
Delayed Memory average *Z*-score	.10 (.64)	− 1.50 (.63)	− 2.49 (.44)	*F*(2,92) = 102.84, *p* < .001
Hippocampal volume (% of whole brain)	.0050 (.0006)	.0046 (.0007)	.0038 (.0006)	*F*(2,95) = 30.54, *p* < .001

Means (SD) are shown for continuous variables; proportions and percentages are shown for sex, race, and current medications. One-way ANOVAs and Fisher’s exact tests were used to compare the groups. Sample sizes for the AD group were smaller for some comparisons because of the inability of some AD patients to complete the measure

### 2FA binding among the diagnostic groups

#### Voxel-based analysis (SPM)

**Fig. 1:**
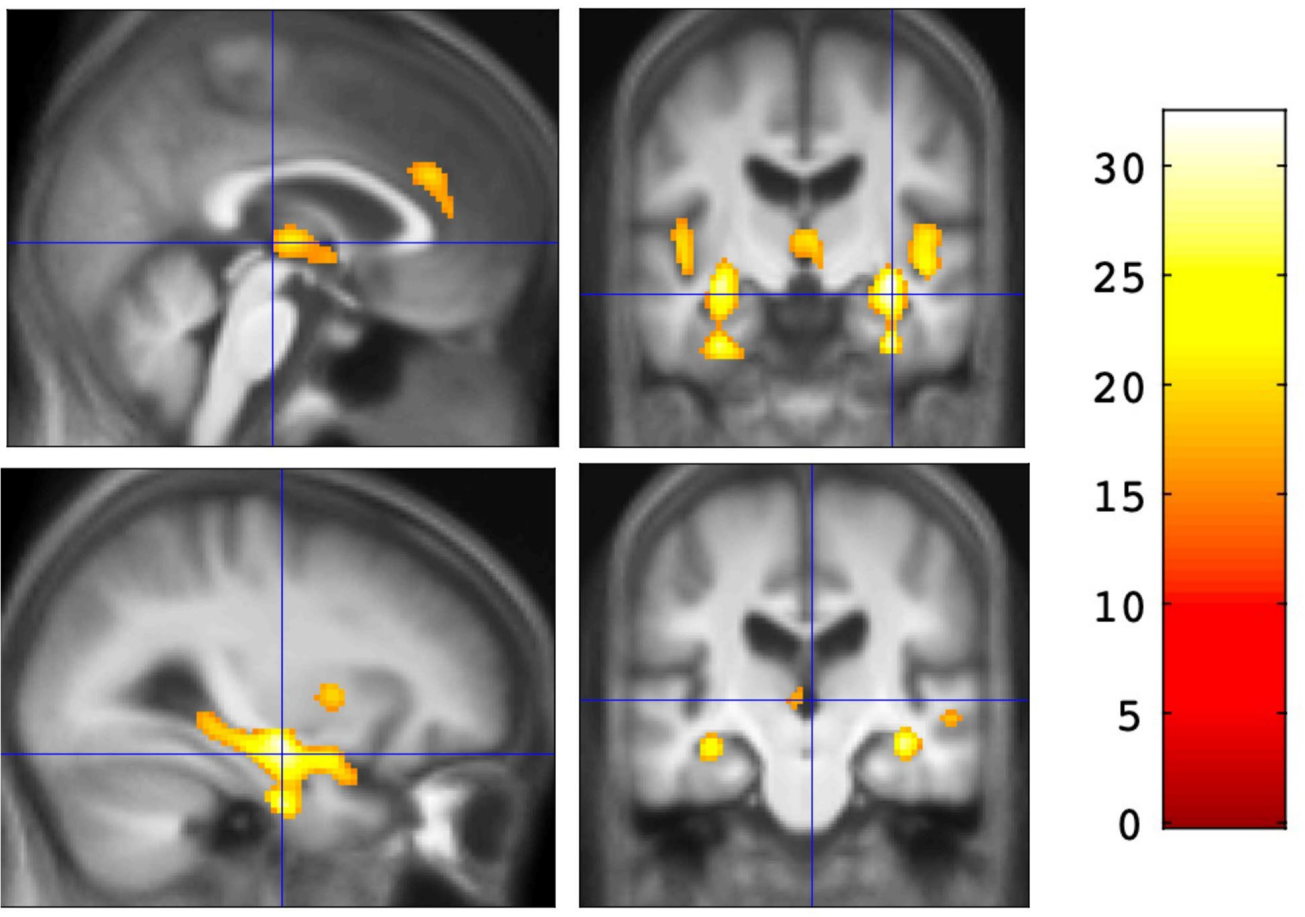
Results of the overall ANOVA showing the effect of diagnosis on 2FA binding (*V*_*T*_/*f*_*p*_ values, *p* < .05, FWE-corrected at the voxel level). *F*-score is shown on the color scale

**Fig. 2:**
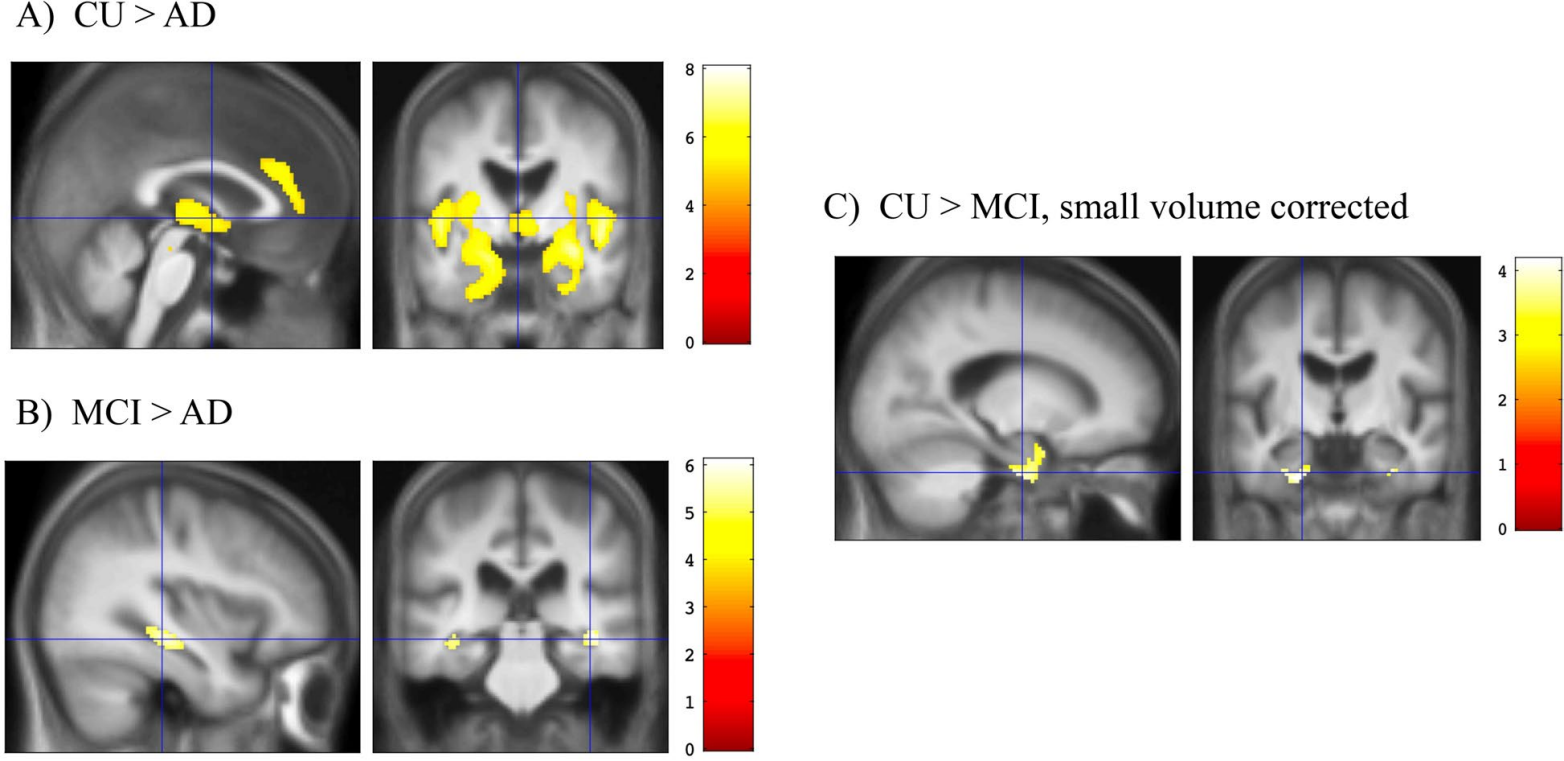
SPM maps of pairwise comparisons of 2FA binding (*V*_*T*_/*f*_*p*_ values). *T*-score is shown on the color scale. For CU > AD (**A**) and MCI > AD (**B**), the results are shown across the entire brain (*p* < .05, FWE-corrected at the voxel level). For CU > MCI (**C**), the results are shown within the bilateral hippocampal/entorhinal mask (*p* < .05, FWE-corrected using small volume correction)

The overall ANOVA of 2FA binding among the three diagnostic groups showed an effect of diagnosis on binding in the bilateral entorhinal cortex, hippocampus, insula, anterior cingulate, thalamus (anterior, ventral anterior, and dorsomedial nuclei), and basal ganglia (including bilateral caudate and anterior putamen) (Fig. 1, Table 2). An independent samples *t*-test showed that AD participants had lower 2FA binding than CU participants in most of the same regions identified in the overall ANOVA (Fig. 2A). Pairwise comparisons also showed that AD participants had lower 2FA binding in the bilateral hippocampus compared to those with MCI (Fig. 2B, Table 3). There were no voxel clusters with binding differences between the MCI and CU groups at the prespecified statistical level when the search volume included the whole brain. However, the results using the small volume correction showed lower binding in the bilateral entorhinal cortex in MCI compared to CU (Fig. 2C; Table 3). There were no voxel clusters in which AD participants showed significantly greater binding than those in the CU or MCI groups. There were also no voxel clusters in which MCI participants showed significantly greater binding than those in the CU group.

**Table 2:** Overall voxel-wise ANOVA showing the main effects of diagnosis on 2FA binding (*V*_*T*_/*f*_*p*_)

Regions	***K***	***P***_**FWE-corr**_	***F*** Statistic	Coordinates
R thalamus, R hippocampus, R entorhinal cortex, R insula, R caudate, R putamen	2923	0	32.28	[30, − 10, − 14]
L thalamus, L hippocampus, L entorhinal cortex, L insula, L caudate, L putamen	2127	0	28.56	[− 30, − 14, − 12]
Bilateral anterior cingulate	75	0.003	19.46	[2, 34, 26]
R middle temporal gyrus	21	0.006	18.62	[50, − 24, − 4]

Findings are significant at the voxel level at *p* < .05 FWE-corrected. *K* = number of voxels. Coordinates referenced to MNI space

**Table 3:** Pairwise comparisons of 2FA binding (*V*_*T*_/*f*_*p*_) between the diagnostic groups, voxel-wise analysis

Regions	***K***	***P***_**FWE-corr**_	***T*** statistic	Coordinates
CU > AD				
Bilateral hippocampus, temporal pole, insula, caudate, putamen, thalamus	9285	0	8.03	[30, − 10, − 12]
Bilateral anterior cingulate	238	0	6.2	[2, 34, 26]
Right temporal pole	44	0.003	5.64	[28, 18, − 44]
Left calcarine fissure	34	0.005	5.49	[− 14, − 62, 8]
MCI > AD				
Right hippocampus	143	0	6.09	[40, − 34, − 6]
Left hippocampus	69	0.001	5.84	[− 36, − 34, − 6]
CU > MCI (within the medial temporal lobe only)				
Right entorhinal cortex	7	0.048	3.76	[30, − 10, − 32]
Left entorhinal cortex	69	0.013	4.17	[− 28, − 12, − 34]

*K* = number of voxels. Coordinates referenced to MNI space

Findings for CU > AD and MCI > AD are significant across the entire brain at the voxel level *p* < .05 FWE-corrected. Findings for CU > MCI are significant at *p* < .05 using small volume correction of MTL

#### ROI analysis

**Fig. 3:**
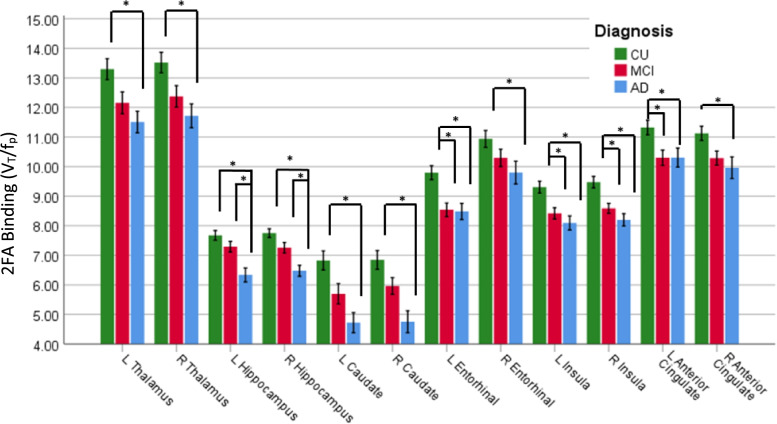
Mean 2FA binding in the core regions of interest in the CU, MCI, and AD diagnostic groups. L, left; R, right. *Pairwise between-group difference, *p* < .05, Bonferroni-corrected

The mean 2FA binding in the six core ROIs (bilateral hippocampus, entorhinal cortex, insula, anterior cingulate, thalamus, and caudate) in the three diagnostic groups are shown in Fig. 3. There was an overall effect of the diagnostic group on all six ROIs (*F*(2,99) = 3.4–15.2, *p*s = .000002–.038). In post hoc pairwise comparisons, participants with AD had lower 2FA binding than those in the CU group in all ROIs, bilaterally (*ps* = .00002-.034, Bonferroni-corrected; *n* = 32 for AD, *n* = 42 for CU). 2FA binding in the AD group was also lower than that in the MCI group in the bilateral hippocampus. 2FA binding in the MCI group was lower than that in the CU group in the left entorhinal cortex, bilateral insula, and left anterior cingulate.

### Effect of medication treatment on 2FA binding

Twenty-three of 32 AD participants and eight of 28 MCI participants were taking a stable dose of AChEI medication at the time of 2FA PET imaging. 2FA binding in the six core ROIs among those taking AChEI medication was compared to those not taking AChEI medication within the AD and MCI groups individually (Table 4). In the AD group, 2FA binding in those taking AChEI medication was lower in the bilateral thalamus and hippocampus compared to those not taking AChEI medication. In the MCI group, there were no significant differences between those on and off AChEI medications in any ROI.

**Table 4:** Mean (SD) 2FA binding (*V*_*T*_/*f*_*p*_) within the AD and MCI groups, for those on and off AChEI medication

Variable	MCI		AD	
	Off ^a^AChEI (***n*** = 20)	On^a^ AChEI (***n =*** 8)	Off AChEI (***n =*** 9)	On AChEI (***n =*** 23)
L thalamus	12.25 (1.76)	11.91 (2.50)	12.96 (1.57)^b^	10.94 (1.98)
R thalamus	12.42 (1.64)	12.28 (2.62)	13.12 (1.53)^b^	11.17 (2.34)
L hippocampus	7.15 (.83)	7.63 (1.20)	7.50 (.84)^b^	5.88 (1.19)
R hippocampus	7.18 (.80)	7.44 (1.30)	7.34 (.82)^b^	6.14 (.93)
L caudate	5.92 (1.62)	5.16 (2.28)	5.40 (1.85)	4.46 (1.91)
R caudate	6.15 (1.52)	5.50 (1.39)	5.90 (1.64)	4.31 (2.11)
L entorhinal	8.55 (1.22)	8.51 (1.28)	8.51 (.87)	8.48 (1.77)
R entorhinal	10.21 (1.35)	10.51 (2.01)	10.13 (1.11)	9.67 (2.48)
L insula	8.22 (.95)	8.93 (1.01)	8.67 (1.16)	7.87 (1.39)
R insula	8.44 (.80)	8.95 (1.07)	8.72 (1.38)	8.00 (1.05)
L anterior cingulate	10.17 (1.20)	10.63 (1.76)	10.71 (1.43)	10.15 (1.93)
R anterior cingulate	10.22 (1.15)	10.47 (1.55)	10.70 (1.43)	9.68 (2.22)

^a^Off and on refers to the current use of cholinesterase inhibitor medication

^b^Significant difference within the diagnostic group, *p* < .05

When the MANOVA of 2FA binding in ROIs across the groups included AChEI medication use as a covariate in the model, participants with AD had lower binding in the bilateral hippocampus, left entorhinal cortex, bilateral insula, and left caudate, compared to the CU group (post hoc pairwise comparisons, *p*s = .003–.037, Bonferroni-corrected; *n* = 32 for AD, *n* = 42 for CU). Participants with MCI had lower binding in the left entorhinal cortex, bilateral insula, and left anterior cingulate, compared to the CU group (post hoc pairwise comparisons, *p*s = .003–.046, Bonferroni-corrected; *n* = 28 for MCI, *n* = 42 for CU). There were no significant 2FA binding differences between the AD and MCI groups for any region in this model.

We also separately examined the effect of stable memantine treatment on 2FA binding in the AD group. There was no significant binding difference between those taking (*n* = 17) and those not taking (*n* = 15) memantine in any core ROI (*p*s > .38 for all comparisons).

Finally, we examined the potential effect of antidepressant treatment on 2FA binding in each of the diagnostic groups. For all ROIs and all diagnostic groups, there were no significant differences between those taking and those not taking antidepressant medication in any core ROI (*p*s > .14 for all comparisons). Similarly, an overall MANOVA that included antidepressants as a covariate did not change the effect of the diagnostic group on 2FA binding in the six core ROIs.

### Age effects

**Fig. 4:**
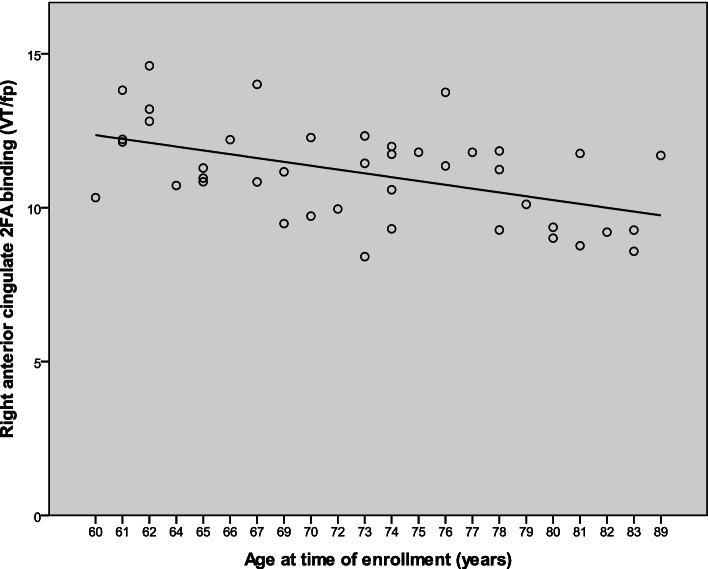
Exemplar scatterplot of the negative association between age and 2FA binding in right anterior cingulate in the CU group

We examined the effect of age on regional 2FA binding in the core ROIs in each diagnosis group. Within the AD group, there was a significant negative correlation between age and 2FA binding in the bilateral hippocampus, left entorhinal cortex, bilateral insula, right anterior cingulate, and left caudate (*r*(30) = − .39–.71, *p* = .000005–.029 for each relationship). Within the MCI group, age was negatively correlated with binding in the right caudate (*r*(26) = − .41, *p* = .03). Within the CU group, age was negatively correlated with 2FA binding in all core ROIs in each hemisphere (e.g., for right hippocampus, *r* = − .50, *p* = .001; *r*s = − .33–.59, *p*s = .00004–.033 for each relationship). As an example, the scatterplot for the relationship between age and 2FA binding in the right anterior cingulate is shown in Fig. 4.

Across all study participants, age was correlated with diagnosis group membership (*r*(100) = − .37, *p* = .00014 with AD = 1, MCI = 2, and CU = 3). To reduce the impact of multicollinearity, the MANOVA was repeated while covarying for age to specifically examine the 2FA binding differences between the MCI and CU groups, as the AD participants were significantly older on average. This analysis, controlling for age, revealed lower 2FA binding in the MCI group compared to the CU group in the left entorhinal cortex and bilateral insula (post hoc pairwise comparison, *p* = .001–.009, Bonferroni-corrected; *n* = 28 for MCI, *n* = 42 for CU).

### Association between ROI 2FA binding and cognition

Across all participants, the Attention average score (ATT) was correlated with 2FA binding in the bilateral hippocampus, insula, thalamus, caudate, and right anterior cingulate (*r*(91) = .23–.34, *p* = .001–.03). The Immediate Recall (IR) average score was similarly associated with binding in the bilateral hippocampus, insula, caudate, and left entorhinal cortex (*r*(97) = .21–.30, *p* = .003–.035 for each relationship). The Delayed Recall (DR) average score was associated with binding in the bilateral hippocampus, insula, thalamus, caudate, and left entorhinal cortex (*r*(94) = .24–.39, *p* = .00009–.042 for each relationship).

Because cognitive assessment scores in the AD group showed floor effects, we repeated the analysis in the combined sample of CU and MCI (*n* = 68). For ATT, correlations were similar to the full sample, although the relationship with 2FA binding in the thalamus was no longer significant and the relationship with the left entorhinal cortex became significant. IR was not significantly correlated with any ROI (*p*s > .18). DR was associated with the left caudate and left entorhinal cortex (*r*(63) = .25–.35, *p* = .003–.038 for each relationship). Covarying for the use of AChEI medication in the analysis of the combined CU and MCI groups did not substantially change the correlations for ATT or IR, while the relationship between DR and binding in the left caudate was no longer significant. Similarly, when covarying for age, IR and DR associations were unchanged, while ATT was positively associated with binding in the bilateral hippocampus and insula, right anterior cingulate, and right caudate.

Finally, we explored whether the inclusion of hippocampal volume in the model would alter the observed relationships between cognitive scores and regional 2FA binding. Of note, there were significant differences in the hippocampal volume between the groups, such that AD had lower volume than CU and MCI (see Table 1, *p*s < .001). There was a marginal difference between CU and MCI (mean difference = .0004, *p* = .06). Within the combined sample of CU and MCI participants and controlling for hippocampal volume, ATT was associated with 2FA binding in the bilateral hippocampus, insula, and right anterior cingulate (*r*(63) = .26–.35, *p* = .004–.038 for each relationship). There were no significant associations between IR and 2FA binding in any ROI. DR was positively associated with binding in the left entorhinal cortex (*r* = .31, *p* = .01).

## Discussion

The results of the whole-brain analysis in this study demonstrated lower levels of nAChR binding in AD dementia compared to cognitively unimpaired older adults in specific limbic and subcortical brain regions: hippocampus, entorhinal cortex, insula, anterior cingulate, thalamus, and caudate/anterior putamen. The complementary ROI analysis of select brain structures generally corroborated these findings and showed that nAChR binding was lower in AD by as much as 30% in the bilateral caudate and 16–18% in the bilateral hippocampus, with smaller reductions in other regions. Regional receptor binding in MCI participants was midway between binding levels in cognitively unimpaired and AD participants. In pairwise comparisons, MCI participants had lower binding than CU participants in the entorhinal cortex, as well as lower binding in the insula and left anterior cingulate in the ROI analysis. AD participants had lower binding than those with MCI in the bilateral hippocampus.

While this study was cross-sectional and cannot address longitudinal change within individuals, the results suggest that the number or binding affinity of nicotinic cholinergic receptors declines initially in the entorhinal cortex and other limbic structures as cognitive deficits appear in the AD process. The vulnerability of the entorhinal cortex to early cholinergic receptor loss may be related to its prominent functional connection to the basal forebrain cell groups [4]. Neurodegeneration in the nucleus basalis of Meynert (NbM) occurs early in the AD process and may precede entorhinal pathology. For example, smaller NbM volume was shown to predict greater degeneration in entorhinal and perirhinal cortices over time, and medial temporal lobe degeneration followed [12]. Similarly, in cognitively unimpaired older adults with cerebrospinal fluid evidence of cortical beta-amyloid, basal forebrain atrophy occurred prior to entorhinal cortex volume loss, which became apparent when cognitive deficits emerged [26]. The functional relationship between basal forebrain and medial temporal lobe structures was also supported by the observation that hippocampal vesicular acetylcholine transporter binding on PET imaging was lower in basal forebrain knockout mice than in wild-type mice [56]. Overall, prior findings and the results of our study support the early involvement of entorhinal pathology in the AD process, and other recent studies suggest that such pathology may be a consequence of basal forebrain degeneration that disrupts entorhinal cholinergic receptors with adverse cognitive consequences. The role of cholinergic receptors within the entorhinal cortex specifically is supported by our finding that deficits in the delayed recall were associated with lower 2FA binding in the left entorhinal cortex in the group of cognitively healthy and MCI participants. This finding persisted with control for the effect of hippocampal volume.

Our study also found that preserved cholinergic receptor binding in the hippocampus distinguished the MCI group from those with AD dementia. While medial temporal cortex structures may be susceptible to early cholinergic receptor decline in the AD process, continued loss of cholinergic receptor density or binding affinity in the hippocampus may contribute to cognitive and functional decline in the progression to AD dementia. The later and enduring impact of hippocampal cholinergic dysfunction may be due to its afferent input from the medial septum and vertical limb of the diagonal band of Broca in the basal forebrain, rather than from the nbM which is affected first in AD degeneration [4, 57].

2FA binding in AD participants taking acetylcholinesterase inhibitor medication was 15–20% lower in the hippocampus and thalamus than that among those not taking AChEI medication. Lower 2FA binding may be due to the competitive inhibition of radiotracer binding at the α4β2 receptor site as a consequence of higher synaptic acetylcholine levels in those taking this medication [58, 59]. However, chronic treatment with AChEI medication may alter the receptor binding affinity over time and lead to lower 2FA activity via a mechanism other than local neurotransmitter competition. Moreover, clinical benefit with AChEI medication may also be due to effects beyond increased synaptic acetylcholine, such as reduced amyloid toxicity or inflammation. In fact, a 2FA PET neuroimaging study found that cognitive improvement with galantamine treatment was not associated with a change in α4β2 receptor availability with treatment [60]. Additional work is needed to better understand the impact of altered cholinergic receptor binding at the molecular, cellular, and neural system levels and the response to pro-cholinergic treatments. While the limited sample size and non-random decisions to treat AD participants with AChEI medication in our study limit the conclusions that can be drawn, the effect of treatment on receptors in the hippocampus and thalamus suggests that these structures may play a distinct mechanistic role in cholinergic treatments. Cholinergic receptor imaging may aid effective drug development to alter AD progression or improve cognitive symptoms by demonstrating cholinoceptive target engagement as well as longitudinal treatment effects linked to cognitive stabilization or improvement.

Interestingly, the results of this study revealed a significant effect of age on nAChR binding in each of the diagnostic groups. In particular, within the cognitively unimpaired older adults, greater age was associated with lower 2FA binding in each of the brain regions that were also affected in AD. Age accounted for as much as 35% of the variance in 2FA binding in this group. This finding suggests that nicotinic receptor density may decline steadily in cognitively healthy adults after age 60 years, although this requires confirmation in a longitudinal study. Similar to our result, Sihver et al. [61] demonstrated lower nAChR binding with age in the medial temporal cortex in healthy elderly participants, and Lagarde et al. [62] found fewer nAChR binding sites in the overall cortical gray matter in older healthy adults compared to younger healthy adults. However, other studies have shown mixed findings regarding cholinergic system decline with age [63]. If nicotinic cholinergic receptor density or affinity declines with age, as suggested by our study, it potentially represents a key mechanism or correlate of other important processes that drive mild memory and other cognitive difficulties that develop with age in healthy older adults. Such age-related cholinergic neuroreceptor alterations may also contribute to later-life vulnerability to superimposed AD pathophysiologies or those of other neurodegenerative disorders. Interventions to preserve cholinergic tone in later life may reduce such vulnerability.

The results of this study that included the largest CU, MCI, and AD samples studied to date in cholinergic imaging studies are consistent with prior neuroimaging work demonstrating lower cholinergic receptor binding in AD [33, 34, 38, 62, 64]. However, the binding levels in MCI, the specific brain regions affected in either AD or MCI, and the relationships with cognition have been inconsistent across studies. The current study adds to the evidence that cholinergic receptor alterations occur in the medial temporal cortex and other limbic regions early in the degenerative process. The study also reveals that the extent of cholinergic receptor binding loss in these regions is modestly associated with a decline in cognitive measures of attention, immediate memory, and delayed memory across the entire group studied. In the subgroup of CU and MCI participants, relationships were strongest between 2FA binding and measures of attention that valued processing speed. Thus, there appears to be an early clinical impact of mild cholinergic receptor dysfunction, most prominently in the domain of attention. Associations between individual cognitive domains and 2FA binding were generally independent of hippocampal volume, indicating that lower neuroreceptor density or affinity contributed to the decline in attention and memory beyond the consequence of hippocampal cell loss. However, concurrent effects or interactions with other pathologies not measured in this study cannot be ruled out.

While this study measured binding at α4β2 nicotinic receptors specifically, other cholinergic receptor subtypes such as α7 nicotinic and M1–M5 muscarinic are also involved in healthy cognition and its decline in degenerative conditions. These additional receptor subtypes contribute to attention, memory, and hippocampal circuit integrity; have bidirectional relationships with other neuropathologies of AD; and are considered potential treatment targets [1, 65, 66]. In concert with α4β2 receptors, the integrity and function of other cholinergic receptor subtypes and their collective effects on additional neurotransmitter systems are relevant to the cholinergic hypothesis of AD.

How these neuroreceptor alterations are linked to traditional pathologies of AD such as β-amyloid and phosphorylated tau in the pathogenic cascade and expression of clinical symptoms of AD remains unclear. Current key issues include the specific sequence of pathophysiologic events in AD, the time course of the sequence, and the critical interactions among etiologic factors in the transition from healthy aging to clinical AD. Because this study did not assess for the presence of β-amyloid, phospho- or total tau, neuroinflammatory alterations, or microvasculature changes, such relationships cannot be addressed. However, the results indicate that reduced cholinergic receptor binding occurs as part of the aging process, at least after age 60, and may contribute to AD vulnerability or may be a specific early AD pathology. However, we cannot rule out the presence of β-amyloid or other pathologies in the elderly CU participants in this study. Another research indicates that (1) β-amyloid deposition and abnormal tau processing occur very early in the AD continuum [67, 68]; (2) elevated CSF p-tau/β-amyloid ratio may drive longitudinal atrophy of nbM and subsequent volume loss in the entorhinal cortex [12], potentially via reduced cholinergic tone; (3) the Ch4 region of the basal forebrain shows a very early accumulation of phosphorylated tau [4]; and (4) early β-amyloid deposition may contribute to basal forebrain degeneration due to the structure’s high sensitivity to amyloid-related loss of trophic factors [69]. Thus, while the decline in limbic nicotinic cholinergic receptor binding may be an early primary event in the AD process, other pathologies also emerge early and there are prominent bidirectional interactions. Longitudinal studies that assess several candidate pathologies, including cholinergic alterations, over an extended time period are needed to better define the path that leads to the cognitive and neuropsychiatric symptoms of clinical AD and to help identify intervention targets.

This study addressed cholinergic receptor deficits in AD specifically, but there are also cholinergic disturbances in other neurodegenerative conditions, such as Parkinson’s disease (PD) and dementia with Lewy bodies (DLB). A small number of comparative post-mortem studies suggest that differences in cholinergic disturbances may distinguish these neurodegenerative conditions. For instance, loss of choline acetyltransferase appears to be greater in DLB compared to AD [70], especially among DLB patients exhibiting hallucinations [71]. Cholinergic neuronal loss is also more severe in the basal ganglia in PD and DLB compared to AD [72], and there is a greater loss of nicotinic receptor binding in the striatum in PD compared to DLB and AD [73]. Yet, a study that employed I-5-Iodo-3-[2(S)-2-azetidinylmethoxy] pyridine single-photon emission computed tomography (SPECT) imaging found that regional α4β2 cholinergic deficits in a pooled AD/DLB sample correlated with the subsequent decline in executive function [29]. The effect was similar in the two diagnostic groups, suggesting that relationships between cholinergic function and executive skills may be similar in the two disorders. Further research can address cholinergic system integrity and respective relationships with individual cognitive domains across neurodegenerative conditions.

## Limitations

This study has several strengths, including a sample sufficiently large to detect moderate group differences in regional cholinergic receptor binding, inclusion of carefully characterized participants, and use of structural MRI in addition to 2FA imaging to assess binding in anatomically defined brain regions and to allow the inclusion of hippocampal volume in evaluating the relationships with cognitive skills. The study also has several limitations. Amyloid biomarkers were not measured, so we were unable to apply an A/T/N framework to the sample. However, the lower hippocampal volumes measured in the study were consistent with those expected in MCI due to AD and in AD dementia, and NIA/AA clinical Alzheimer’s disease criteria were applied by experienced investigators using detailed neuropsychological assessments. Nonetheless, we cannot assume that the MCI and AD participants had elevated cortical amyloid levels, and the results of the study may also apply to those with cognitive deficits resulting from other etiologies. Also, cerebrovascular changes were not included in the models, which could confound the findings. However, structural neuroimaging studies were used to exclude any participant with substantial small vessel cerebrovascular disease or cortical infarct. The MCI and AD groups included predominantly men, and thus, we were unable to assess the effect of sex or a sex × diagnosis interaction. Finally, the modest sample size limited our ability to fully dissect the effects of older age in the AD group, medication treatment, or binding in specific brain regions on the magnitude of cholinergic receptor binding across the groups or relationships with cognitive skills.

## Conclusions

Overall, the results of this study support a more contemporary view of the “cholinergic hypothesis” of AD, demonstrating reduced cholinoceptive binding in specific limbic and subcortical regions in AD dementia that was also present in some brain regions at the MCI phase. Reduced binding was associated with deficits in attention and recent memory in the overall sample and with lower attention skills in the combined group of cognitively healthy and MCI participants. Among cognitively unimpaired older adults, cholinergic receptor binding appears to decline with age and may represent a mechanism for cognitive aging or confer vulnerability to additional neurodegenerative processes in later life. Further studies can help refine the understanding of these relationships, can reveal regional changes over time and their links to other pathologies, and may help identify opportunities for symptomatic or disease-modifying AD treatments.

## Data Availability

Data composites that support the findings of this study are available from the corresponding author upon reasonable request and institutional approval.

## Abbreviations

AD: Alzheimer’s disease
MCI: Mild cognitive impairment
CU: Cognitively unimpaired
AChEI: Cholinesterase inhibitor medication
2FA: 2-[^18^F]fluoro-3-(2(S)azetidinylmethoxy)pyridine
ATT: Attention average index
IR: Immediate recall average index
DR: Delayed recall average index
